# Assessing the Acceptance of and Compliance With Centchroman Usage, a Non-hormonal Contraceptive Pill, Among Postpartum Women: A Cross-Sectional Study at a Tertiary Care Centre in North India

**DOI:** 10.7759/cureus.103611

**Published:** 2026-02-14

**Authors:** Pooja Yadav, K Aparna Sharma, Pragati Singh, Nidhi Bhatt, Vidushi Kulshrestha, Priya Karna, Neena Malhotra, Ashish Bhatt

**Affiliations:** 1 Obstetrics and Gynaecology, All India Institute of Medical Sciences, New Delhi, New Delhi, IND; 2 Public Health Sciences, WHO Country Office for India, New Delhi, IND

**Keywords:** centchroman, non hormonal contraceptive, once weekly pill, ormeloxifene, postpartum

## Abstract

Introduction: Centchroman is a non-hormonal oral contraceptive available in India through the national family planning program under the brand name "Chhaya." As the first non-hormonal oral contraceptive, it inhibits implantation without affecting ovulation or the hormonal axis, making it particularly safe for breastfeeding women. Despite these advantages, its uptake remains limited. This study aimed to assess the acceptance, satisfaction, compliance, continuation, side effects, and failure rate among centchroman users who adopted it as a contraceptive method.

Materials and methods: A cross-sectional descriptive study was conducted through telephonic follow-up of postpartum centchroman users over two years (January 2023 to December 2024) at the Family Planning Clinic, Department of Obstetrics and Gynaecology, AIIMS, New Delhi. A total of 241 women were included. Data on demographics, contraceptive history, side effects, and continuation were analyzed using SPSS v21.0 (IBM Corp., Armonk, NY).

Results: Of 241 users, 72% were postpartum, and 67% were primiparous. The mean age was 28.2 years. While all participants used centchroman for at least one month, 58% continued for 12 months or more (95% CI: 51.7-64.3%). Side effects were reported by 18%, most commonly delayed menses (10.7%). Discontinuation was highest in the first three months, mainly due to access issues (12%) and adoption of alternate methods (12%). Two pregnancies occurred due to missed doses, resulting in a Pearl Index of 0.42 per hundred woman-years (HWY) (95% CI: 0.05-1.52). Overall, 93% of users reported satisfaction, and 88% were willing to recommend the method.

Conclusion: Centchroman demonstrated high acceptability, satisfaction, and a low failure rate among postpartum women, reaffirming its role as a reliable non-hormonal oral contraceptive. Strengthening early counseling and ensuring consistent availability may improve compliance, continuation, and wider utilization within national family planning programs.

## Introduction

India was the first country in the world to launch a National Programme for Family Planning in 1952 and has undergone several transformations over time [1]. The focus of family planning in India has shifted from population control to promoting maternal, child, and newborn health. Despite the strides made in this area, the unmet need for contraception is 9.4% and 3.6% for spacing methods, respectively, according to the recent National Family Health Survey (NFHS-5) [2]. The immediate postpartum period provides a vital opportunity to promote uptake of contraception and to ensure that the woman has the opportunity to start contraception before her first ovulation. However, the choices during this period are limited and must be considered carefully [1].

Oral contraception is a reliable, convenient, and widely used method for limiting unintended pregnancies. Both combined oral contraceptive pills (estrogen-progestin) and progestin-only pills (minipills) are effective methods of contraception; however, progestin-only pills are considered safer in the postpartum period [3]. In 1967, the Central Drug Research Institute (CDRI), Lucknow, India, synthesized a non-steroidal, once-a-week, oral contraceptive pill, centchroman (ormeloxifene). The drug was approved for marketing in 1991. In April 2016, the Ministry of Health and Family Welfare, India, introduced the drug, free of cost, in the National Family Planning Programme with the name “Chhaya” [4]. It is non-hormonal, taken once a week, and acts as a selective estrogen modulator (SERM) and has weak estrogenic and potent anti-estrogenic properties. It has an anti-estrogenic effect on reproductive organs and an estrogenic effect on bones. It prevents pregnancy by inhibiting implantation with no interference with ovulation and the hypothalamic-pituitary-ovarian axis [4,5]. It is free from steroidal side effects, including carbohydrate metabolism, lipid metabolism, blood pressure, and coagulation factors, and hence a safe option for women who cannot take steroidal pills [6]. It is safe during breastfeeding and is not associated with the side effects commonly seen with hormonal contraceptive pills [7,8].

Despite these benefits, its uptake and recognition beyond India have remained low, with limited studies on its acceptance and continuation during the postpartum period [4-8]. The goal of the present study is to evaluate and assess the acceptability, compliance, safety, and effectiveness of centchroman among postpartum women at a tertiary care facility in North India. This study adds longitudinal, real-world evidence on centchroman use using structured telephonic follow-up. It identifies access-related barriers, rather than side effects, as the primary cause of discontinuation, highlighting key programmatic gaps in postpartum family planning services.

## Materials and methods

Study design

This was a hybrid observational study comprising a cross-sectional component to assess acceptability and satisfaction, and a longitudinal follow-up component to evaluate compliance, continuation, side effects, and failure rates among centchroman users, conducted over a period of two years, from January 2023 to December 2024.

Study setting

The study was carried out at the Family Planning Clinic, Department of Obstetrics and Gynaecology, All India Institute of Medical Sciences (AIIMS), New Delhi, a tertiary care center in North India. Institutional Ethics Committee approval was obtained.

Study objectives

The objective of this study was to assess the acceptability and compliance of centchroman among postpartum women at a tertiary care center in North India. Secondary objectives included evaluating continuation rates, side-effect profile, user satisfaction, willingness to recommend the method, reasons for discontinuation, and failure rate.

Study population

Using consecutive sampling, women aged 18-40 years who delivered at or beyond 20 weeks of gestation and accepted centchroman as a postpartum contraceptive method were included. Initiation occurred during the immediate postpartum period (prior to discharge, mostly within one week), and only women who consented and were available for telephonic follow-up between January 2023 and December 2024 were enrolled.

Women aged more than 40 years and those with a history of recent jaundice, chronic infections (including tuberculosis), polycystic ovarian disease, chronic liver disease, chronic kidney disease, hepatitis B or C infection, malignancy, or severe allergic conditions were excluded. Women who could not be contacted due to incorrect contact details or were unable to comply with follow-up because of residence in remote areas were also excluded.

Data collection and follow-up

Of the 900 postpartum women screened, 352 were excluded. Among the 548 eligible women, 307 did not participate further because they either declined centchroman, chose no contraception or an alternative method, or were unreachable by telephone. All eligible women received standardized counseling regarding centchroman, including its dosing schedule, mechanism of action, benefits, and possible side effects. A one-month supply of the drug was provided at the time of discharge, and participants were instructed to initiate the medication immediately as per the recommended regimen.

Follow-up was conducted telephonically using a structured questionnaire. Data collected included sociodemographic characteristics, obstetric and contraceptive history, knowledge and usage patterns of centchroman, compliance, continuation, side effects, and overall satisfaction with the method.

Outcome measures

The outcomes assessed were acceptance, patient satisfaction, compliance, continuation rate, side effects, and failure rate. Failure was defined as the occurrence of pregnancy during centchroman use.

Statistical analysis

Data were entered into Microsoft Excel (Microsoft Corp., Redmond, WA) and analyzed using the Statistical Package for the Social Sciences (SPSS) version 21.0 (IBM Corp., Armonk, NY). Categorical variables were summarized as frequencies and percentages, while continuous variables were expressed as means.

## Results

The mean age of users was 28.2 years (range: 18 to 39 years), with the majority aged between 24 years and 34 years (Table 1). Most users were primiparous (67%) (Table 2). In around 72% of women, centchroman was initiated in the postpartum period, 26% during the interval period, and 2% post-abortion (Table 3).

**Table 1:** Age distribution among centchroman users (n=241)

Age distribution	Centchroman users	Percentage (%)
Under 19 years	2	0.8
19-24 years	39	16.2
24-29 years	94	39
29-34 years	86	35.7
35-39 years	17	7.1
>39 years	3	1.2

**Table 2:** Distribution according to parity (n=241)

Parity	Centchroman users	Percentage (%)
Nulliparous	21	8.7
1	162	67.3
2	50	20.7
3 or >3	8	3.3

**Table 3:** Distribution according to the time of start among centchroman users (n=241)

Time of start	Centchroman users	Percentage (%)
Postpartum	174	72.2
Interval	62	25.7
Post-abortal	5	2.1

Around 80% (191 women) were not using any contraception before centchroman; their stated reason was to achieve conception or the use of natural contraceptives. Out of 20% (N=50) women who had ever used contraception before using centchroman, the majority were using barrier contraception (70%), followed by intrauterine devices and combined hormonal pills.

**Figure 1:**
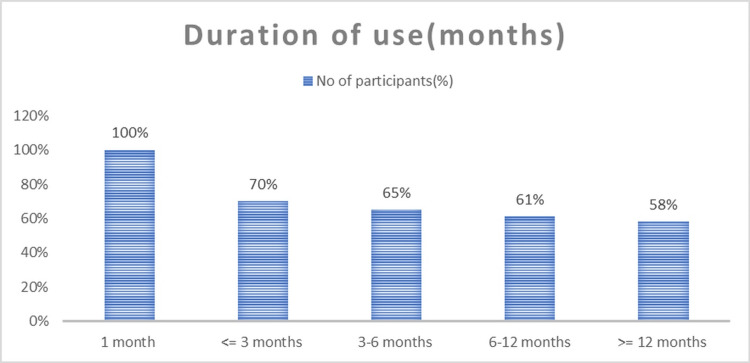
Duration of centchroman use among acceptors

All centchroman acceptors used it for at least one month, 70% up to three months, and 58% for 12 months or more. The discontinuation rate was highest within the first three months of usage (73%), as shown in Figure 1. Most side effects were also reported in the first three months. Nearly all users (99.6%) received pills from the hospital; a few (2.5%) accessed nearby dispensaries.

No serious side effects were reported. Around 44 centchroman users (18%) reported minor side effects, as shown in Table 4. Most of the side effects occurred within three months of usage.

**Table 4:** Side effects among centchroman users (n=241)

Side effect	Frequency	Percentage
No side effects	198	82.2%
Delayed menses	26	10.7%
Headache	5	2.1%
Nausea	5	2.1%
Abdominal discomfort	4	1.65%
Breast tenderness	2	0.83%
Weight gain	1	0.42%

Around 93% of users were satisfied (gave a score of excellent or good), and 88% would recommend it to others. About 96% understood the dosing schedule, and 85% used alarms for adherence.

Out of 241 women studied, two became pregnant due to user failure, where they missed the pills; the Pearl Index was 0.42 HWY.

Around 61% women (N=147) reported current use of centchroman, and 94 women discontinued the drug. The common reasons for discontinuation were access issues and alternate methods (12% each), followed by staying away from their partner (7.5%), side effects (2.5%), and desire for conception (2.1%), as shown in Table 5.

**Table 5:** Reason for discontinuation of drug among centchroman users

Reasons for discontinuation	Frequency	Percentage
Distance from the facility and difficulty obtaining the drug	29	12%
Adoption of alternative contraception (permanent method, LARC, DMPA)	29	12%
Temporarily staying away from their partner because of local cultural beliefs	18	7.5%
Non-compliance	5	2.1%
Desire for conception	5	2.1%
Failure of contraception	2	0.8%
Side effects	6	2.5%

Around 29% (n=29) of the women who stopped the pill at one month reported that they had used up the strip given at the time of discharge and were unable to follow up due to the distance from the medical facility.

## Discussion

This study shows centchroman’s good acceptability among postpartum women. The majority were young, lactating mothers who are an ideal group for centchroman due to its safety during breastfeeding. Delayed menses was the most common side effect, consistent with prior studies (8-26%) [6-7,9-15]. The Pearl Index of 0.42 HWY aligns with published efficacy (0.2-2.0), and the low failure rate suggests high effectiveness when used correctly [3,7,9-11,14-15].

In the present study, the mean age of centchroman users was 28.2 years (range 18-39 years), with the majority of women in the age group of 24-34 years, comparable with other Indian studies [6,9-11]. Women over the age of 40 were excluded from the study. The majority of our acceptors were lactating, and there were no side effects observed in the newborn, as validated by Gupta et al. [12] and Paliwal et al. [8]. The fact that a very small amount of centchroman gets secreted in the breast milk makes centchroman a promising candidate for contraception during lactation [8,12]. The satisfaction rate (93%) mirrors findings from other studies [7,11]. Most users could manage the weekly regimen with digital reminders. However, discontinuation due to access issues, especially after using up the discharge pack, highlights the need for improved drug availability and counseling.

The drug is both safe and effective and is included in the National Family Planning Programme [3]. Around 82% of users had no side effects. Menstrual complaints, especially delayed menses, were the most common side effects among centchroman acceptors, similar to what was reported in the literature. Delayed menstrual cycles were reported in around 10.7% users, which supports the existing literature, ranging from 8-26% [6-7, 9, 11-15]. Systemic side effects were very few and were comparable to the incidence in the general population [3]. The Indian national guidelines mention menstrual delay among 8% of centchroman users [3] and do not mention any other side effects.

The present study shows the efficacy of 99.5%, with two pregnancies due to user failure (Pearl index: 0.42 HWY). This is similar to the National Manual of Oral Contraceptive Pills (efficacy rate of 98-99%) [3], and Miuli et al. [7] having a Pearl index of 0.8 and a low failure rate, compared to Nair et al. [10], Doke et al. [9], and Sarmalkar et al. with a Pearl index of 1.8, 2.0, and 1.0, respectively [14]. In another study by Radhika et al., centchroman had a Pearl index of 0.2 [15], and Gupta et al. reported no failure rate [11]. 

In our study, around 225 (93%) out of 241 centchroman users were satisfied, and 85% of users were able to take the pill regularly. We could only find two studies in the literature on the satisfaction rate among centchroman users. A study by Gupta et al. showed a satisfaction rate of 95% among centchroman users, and the satisfaction rate and continuation rate were similar to postpartum intra-uterine contraceptive devices (PPIUCD) at six months [11]. Another study by Miuli et al. had a satisfaction rate of 94%, which was higher than that of combined oral contraceptives among post-abortal patients. All acceptors used the drug for one month, and 70% for three months. The rate of discontinuation was maximum in the first three months of usage, with major reasons being side effects and accessibility issues. The major reason was the inability to follow up from remote areas (12%), which was even higher in users who discontinued after a month, where they finished the strip provided at discharge and were unable to procure another. Other reasons were temporarily staying away from partner (7.4%) and adoption of alternative contraception (12%). Around 2% users discontinued due to non-compliance, 2% due to side effects, and 2% due to the desire for conception. The high satisfaction rate, coupled with a low side-effect profile, suggests centchroman is well-received when users are properly counselled and have consistent access [7].

The most cited reason for discontinuation, non-availability, highlights the need for bridging accessibility gaps. Low prior contraceptive use among participants points to centchroman’s unique acceptability as a first method. However, gaps in compliance and inconsistent access remain key barriers. Addressing these through improved counselling, follow-up, and supply chain mechanisms could enhance continuation and overall satisfaction. These results support integrating centchroman into postpartum contraceptive counseling. Strategies like antenatal counseling, referral linkages for resupply, and mHealth reminders could reduce discontinuation and enhance continuity.

However, the study highlights critical challenges, particularly related to continuity and accessibility. A significant proportion of discontinuations occurred due to logistical hurdles, such as the inability to obtain refills from remote areas or a lack of structured follow-up. This points to the urgent need to integrate centchroman more robustly within the supply chain, including through primary health centres and community outreach.

Digital health tools such as SMS-based reminders and follow-up calls, along with routine antenatal counseling, can significantly enhance compliance and reduce early discontinuation. Training health workers in centchroman counseling, including information on side effects and proper intake schedule, will further support users. Additionally, the high proportion of first-time contraceptive users in this cohort suggests that centchroman could serve as an effective entry point into family planning programs, particularly among women hesitant to use hormonal methods.

Therefore, patient education in the form of contraceptive counselling during antenatal visits, labor ward admission, and immediate postpartum should be strengthened. Counselling the users to follow up at their nearby healthcare facility for drug procurement will improve the continuation rate. Mobile applications like voice messages for follow-up dates or reminders will help the patient and the healthcare facility keep track.

The study has certain limitations. Being a single-center study conducted at a tertiary care referral hospital managing predominantly high-risk pregnancies, the findings may not be fully generalizable to the general postpartum population or to primary and secondary healthcare settings. The cross-sectional design limits assessment of long-term continuation rates, effectiveness, and delayed adverse effects. Outcomes such as compliance, satisfaction, and side effects were self-reported during telephonic follow-up and are therefore subject to recall and social desirability bias. Additionally, the absence of a comparison group using other contraceptive methods limits direct comparative evaluation of acceptability and continuation. Finally, logistical barriers affecting drug availability may have influenced discontinuation rates and user experiences.

## Conclusions

Centchroman is a safe, effective, and acceptable non-hormonal contraceptive with strong potential for postpartum family planning programs in India. Its once-a-week dosing, minimal side effects, and suitability for breastfeeding women make it particularly advantageous. To fully realize its potential, programmatic efforts should focus on expanding access through consistent supply at peripheral health facilities, improving user education through targeted counseling, and leveraging mobile health technologies to support adherence. Incorporating centchroman into the broader contraceptive method mix with sustained investment in training, monitoring, and community-based promotion can significantly enhance contraceptive choice and autonomy for women in India. Further large-scale, prospective studies are needed to comprehensively evaluate long-term safety, side effects, adherence, and continuation of centchroman under routine programmatic conditions.

## Appendices

Table 6 includes the questionnaire distributed to participants of the study.

**Table 6:** Centchroman questionnaire

A	General Profile	
1	Name of women	
2	Husband’s name	
3	Age	
4	Address	
5	Contact details/Mobile Number	
6	Have you ever attended school?	Yes
		No
7	What is the highest grade you completed?	Uneducated
		Primary
		Secondary
		Graduate and above
8	How long have you been married?	
9	Now I would like to ask about the births you have had during your life. Have you ever given birth?	Yes
		No
10	When did you had last child birth?	Mention month/year
11	How many living children you have?	Mention number
12	Is any of your pregnancy landed in abortion in last 6 weeks	Yes
		No
13	Are you currently pregnant?	Yes
		No
B	Contraceptive Use	
14	Have you ever used any contraceptive in the past?	Yes
		No
15	If yes, what all contraceptives you have used? ((Multiple Response possible and probing may be required)	Condom
		COCs
		Centchroman
		IUCD
		Injectable
		Implant
		Other (please specify)
16	If no, what is the reason of not using any contraceptive?	Wasn’t aware where to get
		Fear of side effects-Infertility
		Cultural taboo and belief
		Dint have money
		Wanted to have family
		Wasn’t required can manage naturally
		Others (please specify)
C	Centchroman use and its awareness	
17	For how long you used/have been using Centchroman (Chhhaya)?	Note response in months
18	When you first started Centchroman?	Immediately after or within 6 weeks of delivery
		Immediately of within 7 days of abortion
		Any period other than the mentioned above
19	Are you currently using Centchroman?	Yes
		no
20	Why you chose this method? (Multiple Response possible and probing may be required)	Only this method is known
		Easy accessibility and Availability
		Easy to switch from other method
		Less known side effect
		Known health benefits
		Accepted by spouse/family
		Ease of Use
		Others (Specify)
21	From where did you get the method? (Multiple Response possible)	From Hospital
		From Nearby Dispensary
		From Shop
		From Health Worker
		From NGO
		Other (please specify)
22	Can you tell how the pill is/was taken?	Daily
		Weekly
		Biweekly for three months and then weekly
		Don’t know/Don’t remember
23	Have you ever missed a pill?	Yes, at one or two instance
		Yes frequently
		No
24	For how long you have usually missed the pill?	few hours to one day
		2-7 days
		>7 days
25	What was the reason for missing the pills?	Difficulty in remembering
		Non availability of pill
		Witnessed side effect
		Other (please specify)
26	What did you do/will do in case you missed the pill?	Take the pill as soon as possible
		Leave the missed pill and take the next scheduled pill
		Discontinue the medicine
		Used emergency contraception
		Used alternative method (other than emergency contraception)
		Contacted health provider
27	Did you follow up with your doctor/nurse?	Yes
		No
28	How many times you have followed up?	Write number
29	At what frequency the follow up was done?	Monthly
		Every 3 months
		Every 4-6 months
		Every 6-12 months
C	Side Effects	
30	Did you had/having any complaints after taking pills?	Yes
		No
31	What health complaints you faced after taking Centchroman pill? (Multiple response)	Weight Gain
		Nausea
		Headache
		Menstrual irregularities
		Increased facial hair
		Irregular periods
		Others
32	After how many months did you faced this health complaint? (encircle 1 for within 3 months; 2 for 3-6 months; 3 for 6-12 months and 4 for after 12 months of use) (probe for all responses given in Q-31)	Weight Gain
		Nausea
		Headache
		Menstrual irregularities
		Increased facial hair
		Irregular periods
		Others
33	What Menstrual changes you witnessed with Centchroman use?	Scanty bleeding
		Oligomenorrhea
		Menorrhagia
		Amenorrhoea
		Any other
34	Did you consult the doctor/seek medical advice for the complaint?	Yes
		No
		Don’t remember
D	Discontinuation	
35	Why did you discontinue the method? (Only Past User)	Wanted to get pregnant
		Got pregnant while on pills
		Side effects
		Wanted another method
		Menstrual irregularities
		Other (please specify)
36	Which method you opted for switching? (In case the response is wanted another method) Only Past User	Dint opt any method
		Barrier method
		COCs
		IUCD
		Implant
		Injectable MPA
		Female sterilisation
		Male sterilisation
		Emergency pill as and when required
		Any other
E	Client Satisfaction	
37	How is/was the overall experience with the chosen method?	Excellent
		Good
		Average
		Bad
		Can’t say
38	Would you like to recommend this method to other couples?	Yes
		No
		Can’t say
39	If No, why you will not recommend	Difficult schedule
		Menstrual irregularities
		Side effects
		Pill is not readily available
		Other (please specify)

## References

[1] (2026). Ministry of Health and Family Welfare, Government of India. National Family Planning Programme: National Health Mission.. https://nhm.gov.in/index1.php?lang=1&level=2&sublinkid=821&lid=222.

[2] (2026). Ministry of Health and Family Welfare, Government of India. Family Planning Division: India’s Vision FP 2020. https://advancefamilyplanning.org/sites/default/files/resources/FP2020-Vision-Document%20India.pdf.

[3] (2026). Family Planning Division, Ministry of Health and Family Welfare, Government of India. Reference Manual for Oral Contraceptive Pills. https://nhm.gov.in/images/pdf/programmes/family-planing/guidelines/Reference_Manual_Oral_Pills.pdf.

[4] Kabra R, Allagh KP, Ali M, Jayathilaka CA, Mwinga K, Kiarie J (2019). Scoping review to map evidence on mechanism of action, pharmacokinetics, effectiveness and side effects of centchroman as a contraceptive pill. BMJ Open.

[5] Kamboj VP, Ray S, Anand N (2018). Centchroman: A safe reversible postcoital contraceptive with curative and prophylactic activity in many disorders. Front Biosci (Elite Ed).

[6] Singh MM (2001). Centchroman, a selective estrogen receptor modulator, as a contraceptive and for the management of hormone-related clinical disorders. Med Res Rev.

[7] Miuli I, Dewan R, Agarwal K (2020). A study to compare acceptability, safety and continuation rates of combined hormonal pill and centchroman as post abortion contraceptives. Int J Reprod Contracept Obstet Gynecol.

[8] Paliwal JK, Grover PK, Asthana OP, Nityanand S, Gupta RC (1994). Excretion of centchroman in breast milk. Br J Clin Pharmacol.

[9] Doke G, Kamda J (2019). A study of Centchroman users with special reference to its contraceptive benefit. Int J Reprod Contracept Obstet Gynecol.

[10] Nair H, Jayasimhan P (2016). A prospective study of centchroman users with special reference to its contraceptive benefit. J Evid Based Med Healthc.

[11] Gupta M, Bansiwal R, Anand HP (2021). Comparison of centchroman and PPIUCD in terms of efficacy, safety and continuation rate in immediate postpartum period. Int J Reprod Contracept Obstet Gynecol.

[12] Gupta RC, Paliwal JK, Nityanand S, Asthana OP, Lal J (1995). Centchroman: a new non-steroidal oral contraceptive in human milk. Contraception.

[13] Agarwal K, Dewan R (2023). Evaluation of acceptability, safety, and continuation rates of centchroman as postabortion nonsteroidal contraceptive pill. Contraception.

[14] Sarmalkar M, Chatterjee M, Mehendale M (2023). Clinical study of centchroman for its contraceptive benefits. Int J Sci Res.

[15] Radhika AG, Suneja A, Malik H, Gupta R (2022). QI initiative to improve utilization of centchroman: a non-steroidal contraceptive. Int J Reprod Contracept Obstet Gynecol.

